# Sediment eDNA metabarcoding reveals the endemism in benthic foraminifera from Arctic methane cold seepages

**DOI:** 10.1093/ismeco/ycaf058

**Published:** 2025-04-02

**Authors:** Ines Barrenechea Angeles, Claudio Argentino, Kristina Cermakova, Maria Holzmann, Jan Pawlowski, Giuliana Panieri

**Affiliations:** Department of Geosciences, UiT the Artic University of Norway, Dramsvegen 201, Tromsø 9010, Norway; Department of Geosciences, UiT the Artic University of Norway, Dramsvegen 201, Tromsø 9010, Norway; ID-Gene Ecodiagnostics Ltd. Plan-les-Ouates 1228, Switzerland; Department of Genetics and Evolution, University of Geneva, Geneva 1205, Switzerland; ID-Gene Ecodiagnostics Ltd. Plan-les-Ouates 1228, Switzerland; Department of Paleoceanography, Institute of Oceanology, Polish Academy of Sciences, Sopot 81-712, Poland; Department of Geosciences, UiT the Artic University of Norway, Dramsvegen 201, Tromsø 9010, Norway; CNR, Istituto di Scienze Polari (ISP), Venezia Campus Scientifico-Università Ca' Foscari Venezia, Via Torino 155, Mestre 30172, Italy

**Keywords:** Arctic methane seepages, benthic foraminifera, microbial mats

## Abstract

Benthic foraminifera are one of the major groups of eukaryotes living at cold seeps on the Arctic seafloor. However, their distribution and endemicity in these habitats have been largely debated. It is still unclear whether foraminiferal species commonly found in cold seeps differ genetically from those in deep-sea environments, and to what extent the seep community is distinct. To address these questions, we analyzed sediment DNA metabarcoding data specifically targeting foraminifera in different deep-water cold seep microhabitats (microbial mats, siboglinid tubeworms field) and reference sites within and outside the seep. Our results revealed microhabitat specificity among benthic foraminifera species. Microbial mats were dominated by a unique type of rDNA sequences assigned to a new lineage of monothalamid (single-chambered) foraminifera not previously reported from any other Arctic location. Other foraminiferal species were found across both seeps and reference stations. This study shows the presence of an endemic benthic foraminiferal species at cold seeps and confirms the existence of many common opportunistic species.

## Introduction

Hydrocarbon seeps along Arctic continental margins [1] emit mainly methane and sulfides creating unique microhabitats that host a specific fauna. These environments are shaped by subseafloor geochemical gradients and bottom water conditions, which influence the distribution of organisms [2–4]. Arctic cold seeps are characterized by two microhabitats: microbial mats and siboglinid forest [5]. Microbial mat areas are characterized by the export of hydrogen sulfide into low oxygen bottom waters, forming the base of the food web and supporting different organisms grazing on bacteria [6]. The siboglinid worms absorb hydrogen sulfide and re-oxidize it into sulfate, which is released into the surrounding sediment and bottom water, maintaining relatively stable oxic conditions [7]. Additionally, the chitinous tubes of siboglinid worms provide a substrate for epibenthic organisms [8].

Foraminifera are significant component of these environments, found in both microbials mats and siboglinid forests. Most studies on cold seep foraminifera focused on hard-shelled species with either calcareous or agglutinated tests. Calcareous foraminifera are frequently found in sulfidic and oxygen-depleted environments such as shallow-water [9,10] and deep-water cold seeps [11,12]. Their taxonomic composition and calcite stable isotopic signatures have been studied to retrace past methane seeps, providing insights into seep activity and intensity [13,14]. These hard-shelled foraminifera serve as valuable proxies for reconstructing methane emissions, enhancing our understanding of methane dynamics and their relationship to climate change events [15,16].

However, significant knowledge gaps remain regarding foraminifera in cold seeps. While endemism has been documented for various organisms inhabiting these environments [17–19], the absence of endemic foraminifera has been consistently reported in all investigations conducted so far [11,20], suggesting that foraminiferal assemblages in cold seeps are predominantly composed of opportunistic species [11,21]. Nonetheless, this conclusion, is derived from studies that primarily focus on hard-shelled foraminifera, resulting in an incomplete perspective on foraminiferal diversity. Morphology-based studies of hard-shelled foraminifera suggest that foraminiferal seep communities are mainly composed of opportunistic species, taking advantage of abundant food sources and ingesting methanotrophic bacteria [20–22]. It has been hypothesized that these opportunistic species may migrate there from surroundings areas (non-seep zones) [20,23]. Up to now, there is no documented evidence of endemic methane seep foraminifera.

Monothalamous foraminifera, with their single-chambered organic or agglutinated tests, have received less attention, despite their presence being reported in Arctic cold seeps such as the Vestnesa pockmarks [11] and the Håkon Mosby Mud Volcano [24]. To date, only limited genetic data on monothalamids from methane seep areas have been published, highlighting the need for further research [25–27]. Additionally, morphology-based identification methods often fail to detect cryptic speciation, potentially obscuring the presence of genetically distinct populations adapted to specific deep-sea ecosystems [28,29]. Therefore, the potential endemism of seep foraminifera cannot be ruled out based on existing studies.

In this study, we conducted a metabarcoding analysis of foraminiferal communities from surface sediments collected with an ROV (Remotely Operated Vehicle) in four deep-water cold seeps, three off the western continental margin of Svalbard (West Svalbard Margin, Vestnesa Ridge and Svyatogor Ridge), and one in SW Barents Sea (Håkon Mosby Mud Volcano). The precise sampling capability of the ROV minimizes disturbance to the surrounding environment, ensuring the reliability of our samples and results. Our study focused on assessing foraminiferal diversity and their distribution across various habitats, both within and beyond the seeps. Additionally, we investigated whether certain foraminiferal species are exclusive to seep environments.

## Materials and methods

### Study area

**Figure 1:**
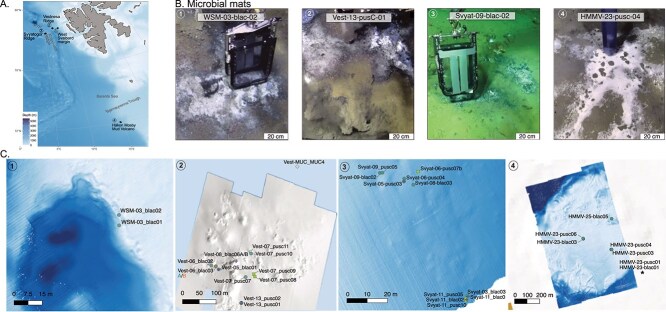
**(**A) Location map of the four deep-water sites sampled during AKMA1 and AKMA2 expeditions. (B) Microbathymetric maps and sampling location within the sites: (1) WSM, (2) Vestnesa ridge. The reference outside (ref. out) samples vest-MUC_MUC1, vest-MUC_MUC2, vest-MUC_MUC3 and vest-MUC_MUC4 are located 2 km N, (3) Svyatogor ridge on the west part of Svalbard. The reference outside (ref.out) samples Svyat-MUC_MUC2_1, Svyat-MUC_MUC2_2, Svyat-MUC_MUC2_3 and Svyat-MUC1_C4 are located 4 km NE and (4) Håkon Mosby Mud Volcano in the Barents Sea, HMMV-24- blac04 and HMMV-24-pusc07 are located 1.3 km NW of the mud volcano. Shapes from sampling stations correspond to different microhabitats (circles: Microbial mats, cross: Tubeworms, asterisks: Gastropods, squares: Reference inside pockmarks (ref.in), diamonds: Reference outside (ref.out). Mixed microhabitats are represented by a circle containing the respective shapes. C. Seafloor images illustrating the precise sampling of microbial mats using the ROV in the four different localities.

The three cold seeps off the western continental margin of Svalbard—(West Svalbard Margin [WSM]), Vestnesa Ridge (Vestnesa), and Svyatogor Ridge (Svyatogor) —are the northern most seeps in the world (Fig. 1A). The area is known to store large quantities of methane in the form of gas hydrates [30,31]. The three ridges lie on either side of the Molloy Transform Fault (MTF) and at the north of the Knipovich Ridge. Vestnesa Ridge is a 100 km long contourite ridge located at east of the MTF. Svyatogor Ridge represents a sedimented ridge with an actively rifting spreading environment, extending 46 km in length, and situated west of the MTF. Along Vestnesa Ridge, several pockmarks are associated with active venting of methane-rich fluids from confined deep-water gas hydrate and free gas reservoirs [32]. Active seeps have been recently reported from Svyatogor Ridge [33], where subsurface gas chimneys and seafloor pockmarks also occur [34]. During the AKMA1 expedition [35], a pockmark at a depth of 900 m on the WSM was sampled (Fig. 1B). Another pockmark located at 1200 m water depth on the southernmost end of Vestnesa Ridge was targeted during the AKMA2 expedition [36] (Fig. 1B). ROV visual inspection of this site revealed, for the first time at this location, gas hydrates exposed at the seafloor [36].

The fourth cold seep examined in this study is the Håkon Mosby Mud Volcano (HMMV). HMMV was discovered in 1990 [37] and is situated in the southwestern Barents Sea, at the termination of the Bjørnøyrenna Trough Mouth Fan (Fig. 1A, B). Located at a depth of 1250 m, the HMMV actively releases a substantial amount of methane sustaining shallow gas hydrates [38–40]. Furthermore, the site exhibits high thermal and geochemical gradients from its center to the margins, creating distinct habitat zones [41].

### Sampling

Sediments and pore water samples were collected using push (pusc) and blade (blac) cores with ROV and multicores (muc) during the AKMA1 [35] and AKMA2 [36] expeditions on RV Kronprins Haakon (Fig. 1C, for more details, Table S1). Environmental DNA (eDNA) and geochemical analyses were carried out on samples taken from the same cores or their twin counterparts. The ROV sampling ensured precise core collection from small areas, such as seafloor mat patches, and minimized sediment disturbance by positioning the ROV against the current before the sampling. Sediment for eDNA analyses were sub-sampled immediately after core collection. Approximately 5–10 g of the top core sediment was collected using a sterile spoon, placed into a sterile vial and preserved in Lifeguard solution at −20°C until DNA extraction.

At each location, we sampled the following microhabitats: microbial mats (mat) and tubeworm forests formed by siboglinid tubes (tub.). Areas where microbial mats overlapped with tubeworms, were referred as “mat-tub”, while those with abundant gastropods were termed “mat-gast”. Additionally, we collected samples from non-seep areas within pockmarks, situated a few meters from seep-associated microhabitats, referring to these as "reference inside" (ref. in). Samples collected several kilometres away from seep areas were designated as “reference outside” (ref.out). Two samples of microbial mats (WSM-03-BlaC-01 and WSM_03_blac02) from WSM were analysed. These samples were situated at the northern end of Vestnesa Ridge and grouped with those from Vestnesa in subsequent analyses. At Vestnesa, samples were collected within a pockmarck ~500 meters in diameter, featuring exposed gas hydrate, active methane bubbling, patches of tubeworms, a mixed habitat (mat-tub and mat-gast), and a reference site inside the pockmark (ref. in). Multicore samples were taken several kilometers away from the pockmark, with no methane influence (ref. out). In Svyatogor pockmarks, cores were taken from an extensive and dense microbial carpet, areas with tubeworms and mixed areas (mat-tub) and reference sites inside and outside the pockmarks, without methane activity or specialized fauna. At the Håkon Mosby mud volcano, cores were taken from dense microbial mats and reference sites located outside the seep area (ref. out). Inside the pockmarks, microbial mats and tubeworms were sampled along a gradient capturing their transition. During the AKMA2 expedition, the top 2 cm of sediment collected by push and blade cores were examined on board for living foraminifera. Selected multi-chambered and single-chambered foraminifera were isolated and preserved for DNA barcoding, carried out subsequently at the University of Geneva. The obtained sequences were submitted to the NCBI foraminiferal database and are included in a separate publication [42].

### Foraminifera DNA isolation from sediments and amplification

A total of 34 samples was collected during the AKMA 1 and AKMA 2 expeditions (Table S1). About 10 g of sediment were extracted from each sample using the DNeasy® PowerMax® Soil Kit (QIAGEN). We have targeted a short foraminifera-specific 37f hypervariable region of the 18S rRNA gene for diversity analyses. Phylogenetic analyses of foraminifera from microbial mat samples are based on the hypervariable regions 37f and 41f of the same gene. The 37f region was amplified using primers 14F1 (5′-AAGGGCACCACAAGAACGC-3′) and s15 (5′- CCACCTATCACAYAATCATG −3′), the amplicon size ranged from 86 to 190 base pairs (bp). For the amplification of the 37f and 41f regions primers 14F1 and s17 (5′-CGGTCACGTTCGTTGC-3′) were used, the resulting amplicon size ranged from 230 to 380 bp [43]. For each extracted sample, a unique combination of tagged primers with eight nucleotides at 5′ end was used for amplification [44,45]. Additionally, three PCR replicates and one negative control were generated for each sample and verified through agarose gel electrophoresis. The three PCR replicates were combined for each sample and quantified using high-resolution capillary electrophoresis (QIAxcel System, QIAGEN). Combined PCR replicates were pooled equimolarly for each multiplexed library and markers (37f and 37f–41f). Each pool was purified using High Pure PCR Product Purification kit (Roche). Sequencing libraries were prepared using TruSeq® DNA PCR-Free Library Preparation Kit (Illumina) and quantified by qPCR using Kapa Library Quantification Kit for Illumina Platforms (Kapa Biosystems). The libraries were paired-end sequenced on a MiSeq instrument using the kit v2 (300 cycles) for libraries containing amplicons of the 37f regions and kit v3 (500 cycles) for libraries with 37f and 41f amplicons.

### Bioinformatics

Raw data analysis was performed using the SLIM pipeline [46]. Briefly, the raw sequences were demultiplexed using Double Tag Demultiplexing. Further steps comprising quality filtering, learning errors, merging and chimera removal were done using DADA2 [47] R package with standard parameters. We obtained an Amplicon Sequencing Variants (ASVs) matrix with counts and sequences. We applied LULU [48] correction and the final filtering steps were as following: removing ASVs with <10 total reads, deleting ASVs with short sizes (< 60 bp), removing sequences without conservative genetic patterns in the 37f region (“GACAG”) (Table S2). For the remaining sequences, we applied a minimum relative abundance threshold, setting to zero the reads of any ASV with abundance below 0.005% of the total reads of this sample. This was done to eliminate the potential tag jumps between samples. The remaining sequences were taxonomically assigned using VSEARCH [49] with a 90% min. Similarity threshold using a local database for benthic foraminifera [50] to which the barcoded sequences of specimens collected during AKMA2 were included (Fig. S1).

### Phylogenetic placement

The obtained sequence of a newly identified monothalamid foraminifera dominating microbial mat samples (*Cold Seep Monothalamid 1* [*CSM1*]), was added to an alignment containing 19 monothalamid sequences publicly available at NCBI database. The alignment was performed using MAFFT v.7 with the L-INS-i method [51] and 1691 sites were utilized for subsequent analysis. A phylogenetic tree was constructed using the maximum likelihood phylogeny (PhyML) method as implemented in ATGC: PhyML 3.0 [52]. An automatic model selection, guided by the Akaike Information Criterion (AIC) through SMS [53], resulted in the adoption of a GTR + R substitution model for the analysis. The initial tree was based on BioNJ, and bootstrap values (BV) were computed based on 100 replicates. The species *Arnoldiellina fluorescens* (HE775247) was designated as the root for the phylogenetic tree.

### Statistics

Planktonic foraminiferan ASVs were removed before statistical analysis took place. All statistical analyses were performed in R Studio using mainly phyloseq [54] and microeco [55] packages which use vegan [56] package for alpha and beta metrics. The ASV table was normalized using Scaling with Ranked Subsampling [57] method before calculating the alpha diversity metrics (richness, Shannon–Wiener indexes (H′) [58] and Inversed Simpson [59]) for each microhabitat and compared between them using one-way analysis of variance. For beta diversity, principal coordinates analysis (PCoA) [60] was performed using Bray–Curtis distances to illustrate similarities between microhabitats. Due to the lack of methane concentration data from microbial mat sites, which limits our ability to confirm the presence of endemic species in high methane flux areas, we relied on sulfate data as an indirect proxy for the depth of the sulfate–methane transition and upward methane fluxes. To demonstrate the effect of sulfate concentration on the most abundant taxa and microhabitats, redundancy analysis (RDA) [60] was performed using values from depths with the lowest sulfate concentration (9 to 16 cm). For bacterial mats, these depths align with the sulfate–methane transition zone (SMTZ). The selected samples, along with their respective sulfate values and depths, are provided in a supplementary table (Table S1).

### Pore-water sulfate concentration

Down-core sulfate concentration in sediment cores is a key parameter to track the position of the SMTZ and indirectly the magnitude of methane fluxes. Pore-water samples were extracted onboard immediately after retrieving the blade and push cores, using rhizons inserted into the pre-drilled holes in the sediment cores. The collected pore-water was stored in sterile syringes and frozen until laboratory analyses. The sulfate concentration was measured via ion chromatography at TosLab AS in Tromsø, Norway, following the NS-EN ISO 10304-1 protocol with an estimated analytical precision of 15% (RSD, Relative Standard Deviation). We modelled the downward diffusive sulfate fluxes (J; nmol cm^−2^ d^−1^) based on the linear part of the concentration profiles entering the SMTZ (in the cores presenting an SMTZ) and in the lowermost part the cores when not presenting an SMTZ. We applied Fick’s First Law (Eq. 1):


(1) \begin{equation*} J=\mathrm{\varphi} \times \mathrm{Ds}\times \frac{dC}{dz} \end{equation*}

where φ is the sediment porosity, Ds the whole-sediment diffusion coefficient and *dC*/*dz* is the concentration gradient [61]. We assumed a constant average porosity of 0.7 and bottom water temperature of ~0°C for the examined sites. Ds was obtained from the free-solution molecular diffusion coefficients of sulfate, Do, obtained from the Marelac [62] package for R and corrected for tortuosity [63] (Fig. S2).

## Results and discussion

### Taxonomic composition

**Figure 2:**
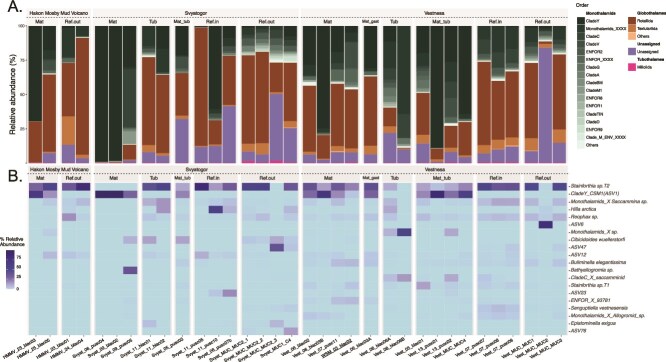
(A) Foraminiferal taxonomic composition at order level of main classes (Monothalamids, Globothalamea, Tubothalamea) from Håkon Mosby Mud Volcano, Svyatogor, Vestnesa, and WSM (underlined in black). (B) Heatmap presenting the 20-topmost abundant taxa in all the investigated microhabitats (seeps areas: Microbial mats, tubeworms, mat-tub, mat-gast and non-seep areas: Reference inside and outside).

At a higher taxonomic level (Fig. 2A), monothalamids dominated seep areas across microhabitats, whereas hard-shelled foraminifera (Globothalamea orders: Rotaliida and Textulariida) were more prevalent in non-seep areas, both in closer proximity (ref. in) and farther away (ref. out) from microbial mats. Tubothalamea was the least abundant class across all sites. The proportion of different taxa varies between sites (Fig. 2A). In Håkon Mosby samples, monothalamids and Globothalamea were dominant, with some unassigned taxa present in lower abundance. Svyatogor samples displayed greater variation among the main foraminiferal orders. Rotaliids and textulariids were more prevalent in reference outside sites (ref. out), while microbial mat samples were composed almost entirely of monothalamid clades.

In Vestnesa samples, mixed microhabitats (mat-tub, mat-gast) showed higher variability with several monothalamid clades being prominent, while rotaliids and textulariids dominated the reference inside (ref. in) and outside (ref. out) samples.

At the species level (Fig. 2B), the most abundant taxon across all studied sites was the rotaliid *Stainforthia* sp. T2. This species dominated both reference and cold seep samples, except in microbial mats where the newly identified new monothalamids lineage, *CSM1* was the most abundant. *CSM1* was exclusively found in microbial mats and environments with shallow SMTZ, less than 10 cm deep (Fig. S2). It was absent in tubeworm samples with deeper SMTZs and reference samples that lacked SMTZs, as indicated by higher sulfate concentrations (Fig. S2). Both *Stainforthia* sp. T2 and *CSM1* co-existed with similar abundances in some transition zones (mixed microhabitats), where small microbial mat patches were interspersed among tubeworms and gastropods (mat-tub and mat-gast). Reference samples inside and outside pockmarks were generally dominated by *Stainforthia* sp. T2, except for two samples from Svyatogor and from Vestnesa where other monothalamids such as Monothalamid_X and *Hilla arctica*, were more prevalent.

Apart from *Stainforthia* sp. T2 and *CSM1*, the most common foraminiferal species included monothalamids such as *Saccammina* sp., *Hilla arctica*, *Bathyallogromia* sp., and *Senguptiella vestnesensis*; rotaliids such as *Cibicidoides wuellerstorfi*, *Buliminella elegantissima*, and *Epistominella exigua*; and the textulariid *Reophax* sp., which was common in reference sites and rarely found in microbial mats. Among monothalamids, unique occurrences were noted in the case of the unassigned ASV6 found in a reference site outside a pockmark on Vestnesa ridge and *Hilla arctica* being present mainly in reference inside and outside samples. The remaining top twenty species included two undetermined monothalamids (Monothalamid X) and unassigned ASVs 12 and 47. These taxa were, present in various microhabitats within cold seeps, including mixed microhabitats.

Our results contrast with morphology-based studies of cold seep foraminifera, such as those by [11], which reported a high abundance of calcareous species, primarily from the order Rotaliida. The authors also identified the multi-chambered agglutinated species like *Reophax* spp. and *Sigmoilopsis* sp., but included only two monothalamous species, *Hyperammina* sp. and *Bathysiphon* sp. Morphology-based studies specifically focused on cold seep monothalamids are rare. However, their high diversity was documented by [24], who described around 20 different morphotypes using terms like “oval” and “grape-shaped” forms, from the Håkon Mosby Mud Volcano sediments. Our study complements this morphological analysis with genetic data, revealing a high diversity of monothalamids in cold seep environments. We identified more than 100 ASVs belonging to different monothalamids clades (clade Y, C, V, etc.) across all sites investigated. Some of these ASVs could be assigned to species (*Hilla arctica*) or classified at the family or genus level (e.g. *Bathyallogromia* sp., *Saccammina* sp.), while many remained unassigned at lower taxonomic levels. This underscores the remarkable diversity and abundance of deep-sea monothalamids, aligninig with previous research [64–66].

### Microhabitat specificity of foraminiferal species

**Figure 3:**
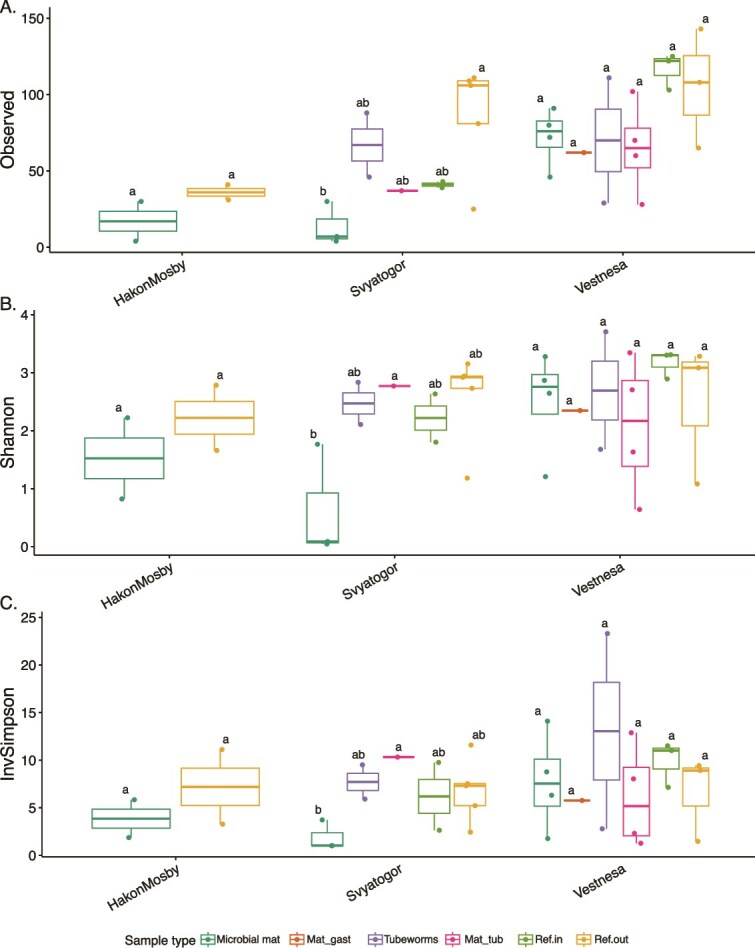
Box plots showing alpha diversities of each site/microhabitat. (A) Observed ASV (richness), (B) Shannon indexes (H′) and (C) Inversed Simpson indexes. Lower-case letters indicate significant differences between microhabitat within sites.

Alpha diversity metrics showed high variability across microhabitats at all sites, particularly in Vestnesa and Håkon Mosby (Fig. 3). However, no statistically significant differences were detected among microhabitats, except at Svyatogor, where microbial mats exhibited significantly lower richness and diversity compared to other non-reference microhabitats. Generally, microbial mats yielded less ASVs (richness, Fig. 3A), lower Shannon (Fig. 3B) and inverse Simpson values (Fig. 3C), indicating that their foraminiferal communities consist of few but dominant taxa. Non-seep areas (ref. in, ref. out) displayed the highest richness and diversity indices, followed by mixed microhabitats (mat-tub, mat-gast) and tubeworm habitats, although the tubeworm zone at Svyatogor had a higher richness and displayed similar diversity to non-seep areas.

**Figure 4:**
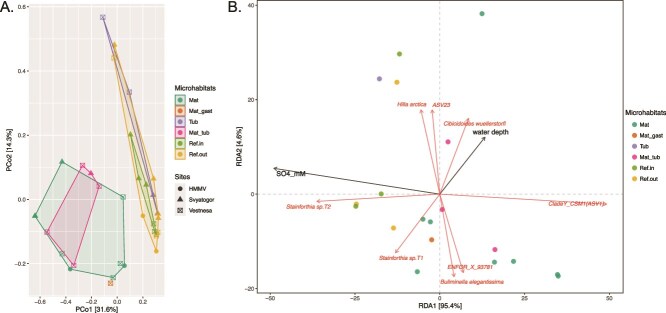
Beta diversity plots. (A) PCoA showing the beta diversity based on Bray–Curtis distances across all sites. (B) RDA showing directionnal arrows representing abundant taxa (ASV) and environmental parameters (SO_4_ and water depth), with microhabitats represented by dots.

The PCA plot (Fig. 4A) revealed that foraminiferal communities inside (ref.in) and outside (ref. out) the pockmark and those associated with tubeworms from different sampling sites, share similar species and clearly differ from microbial mats. Foraminiferal communities from different microbial mats samples are similar to communities from mixed microhabitats dominated by gastropods (mat-gast) or tubeworms (mat-tub) (Fig. 4A).

The RDA plot (Fig. 4B) highlights the relationship between sulfate concentration (lowest value in the profiles, also corresponding to the depth of the SMTZ where intercepted, Fig. S2), microhabitat communities and foraminiferal taxa. Sulfate data is key to track the position of the SMTZ, which is a critical interface in marine sediments where anaerobic oxidation of methane (AOM) occurs [67,68]. This process is significant in cold seep, as it influences the chemical gradients (e.g. H_2_S) that shape microbial and foraminiferal communities. Our pore-water data (Fig. S2) shows that microbial mat microhabitats exhibit high downward sulfate fluxes which indicate high AOM rates and methane fluxes across different study areas [5].

Microbial mat samples are positioned opposite the sulfate vector in our plot, except for a few outliers where sulfate concentrations were high, similar to those in tubeworm environments (Fig. 4B). The sample distribution highlights the association of microbial mats and mixed habitats (mat-tub, mat-gast) with low-sulfate conditions. The presence of outliers may be attributed to potential seawater infiltration after sampling. Sulfate concentration exhibits a positive correlation with reference sites, which are characterized by high sulfate concentration (and indirectly by the absence of methane) as well as with tubeworm habitats, where bioturbation may alter or disrupt sulfate and methane fluxes (Fig. 4B).

These geochemical characteristics suggest that microbial mats across the study areas share similar chemical environments and therefore influence their foraminiferal composition. The foraminiferal assemblage in these microbial mats is dominated by a newly identified monothalamid species belonging to Clade Y and referred as *CSM1*. The species exhibits a strong negative correlation with sulfate, thriving in areas with lower sulfate levels, which indirectly indicates its preference for environments with higher methane production. *CSM1* also appears sporadically in zones with tubeworms and gastropods, but only in areas where visible patches of microbial mats are present (Fig. 4B). Its occurrence correlates with shallow SMTZ sites (~5-10 cm, Fig. S2), suggesting that the species is specifically adapted to sulfidic sediments affected by intense AOM. The absence *CSM1* in reference sites confirms its endemicity.

On the other hand, potentially opportunistic species dominate sites other than microbial mats within the pockmark. Our data indicate that these species are genetically identical to those found in reference sites distant from the cold seeps. Some of them, such as *Stainforthia* spp. and *C. wuellerstorfi* are common calcareous deep-sea species previously reported from cold seep sites [69,70]. Their presence both inside and outside pockmarks suggests a certain level of adaptability to the extreme geochemical conditions, including methane and sulfide exposure, potentially benefiting from the high productivity characteristic of cold seeps. Several studies have identified *Stainforthia* as an opportunistic genus [71], capable of thriving in low oxygen conditions [72] and sulfidic environments [73]. A species within this genus has been observed to sequester chloroplasts and possess abundant peroxisomes, adaptations that may enhance its survival in such harsh conditions [73,74]. However, our study shows that neither *Stainforthia* nor other opportunistic species appear capable of surviving in microbial mats where high sulfide fluxes occur as a result of AOM-related sulfate reduction in the shallow subsurface.

**Figure 5:**
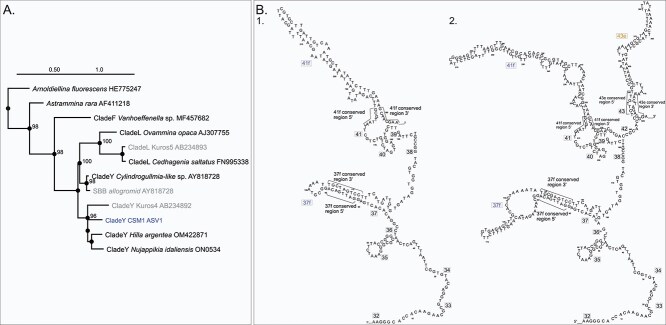
Genetic characteristic of CSM1. (A) Phylogenetic tree of some monothalamids with placement of CSM (long sequence, in bleu) and other sequences found in other cold seeps (in grey). (B) Secondary structure model of partial SSU rDNA (single stranded) covering variable regions 32 to 42 according to [43] and hypervariable regions for foraminifera (37f and 41f) and eukaryotes (43e). 1. CSM1; 2. *Micrometula hyalostriata*.

### The new lineage CSM1 is exclusively associated with microbial mats

The predominant sequence in the metabarcoding dataset was ASV1, corresponding to the novel lineage *CSM1*, which was, identified exclusively in microbial mats. Since no specimens could be isolated and morphologically characterized, and the sequenced fragment was too short for phylogenetic analysis, a longer fragment including the foraminifera-specific hypervariable region 41f of the 18S rRNA gene was analyzed to overcome these limitations. Phylogenetic analysis confirmed the placement of *CSM1* as a new lineage within Clade Y (Fig. 5A). Further characterization of *CSM1* involved comparing its SSU rDNA secondary structure with that of another monothalamid, *Micrometula hyalostriata* [43]. This comparison revealed the absence of the highly variable region 37f in *CSM1*, which is also the case for other Clade Y species [50]. Region 41f, an extension of helix 41, was present in *CSM1*, but was shorter than in *M. hyalostriata* (Fig. 5B).

The identification of *CSM1* as a new lineage within Clade Y exemplifies the adaptation of monothalamid foraminifera to cold seep environments. Monothalamid species have been reported in anoxic and sulfide-rich sediments, such as those in the Santa Barbara Basin (SBB) [25,27,75]. One of these species, referred to as the SBB monothalamid, has been shown to host endobionts that aid survival in harsh environments through sulfur inclusion and nitrogen accumulation [75]. These endobionts, likely sulfide oxidizers or denitrifiers such as Beggiatoa, facilitate the host's adaptation to extreme conditions [25]. However, our phylogenetic analysis (Fig. 5A) indicates that the DNA sequence of the SBB monothalamid differs from that of *CSM1*, suggesting that multiple endemic monothalamid lineages may inhabit cold seeps, with much of their diversity still unexplored. To investigate potential genetic differentiation at the population level of monothalamids, future studies should consider sequencing longer fragments of the 18S gene, as done in a metabarcoding study of foraminifera from Kuroshima Knoll methane seeps [26,76] or employing non-coding regions (ITS) with higher variability as used to test the bipolar distribution of deep-sea foraminifera [77]. Additionally, other genomic techniques, such as metagenomics or transcriptomics could provide insights into the relationships between cold seep monothalamids and their prokaryotic endobionts.

## Conclusion

To conclude, our study provides new insights into the diversity of cold seep foraminiferal communities. It reveals that cold seep foraminifera include both endemic and opportunistic species, with their distribution closely linked to specific microhabitats within the seeps. This relationship is influenced by interstitial fluid chemistry, which reflects the magnitude of subsurface anaerobic oxidation of AOM. The discovery of a new species dominating microbial mats suggests a potential adaptation of certain foraminiferal species to the unique chemical conditions of these environments, characterized by high hydrogen sulfide and low oxygen levels, although it remains unclear whether methane reaches the sediment surface.

The characteristic genetic sequence of this species could serve as proxy for methane seeps in environmental paleogenomic studies of the evolution of the Arctic Ocean.

However, it must be emphasized that metabarcoding data provide only an initial molecular perspective on the ecology of seep foraminifera. Further research is required to isolate and characterize this newly identified seep-specific monothalamid species, particularly in relation to its potential association with bacterial endosymbionts. Further DNA barcoding studies of foraminifera isolated from seeps and the analysis of their genomic and/or transcriptomic data might lead to a better understanding of their adaptation mechanisms. Finally, metagenomic analysis could help explore the interactions between foraminifera and cold seep microbial communities, uncovering the genes responsible for their survival in these extreme environments. This would contribute to a better understanding of their ecological role within these complex ecosystems.

## Supplementary Material

Figure_S1_ycaf058

Figure_S2_ycaf058

Table_S1_ycaf058

Table_S2_ycaf058

## Data Availability

The data generated and analyzed during the current study are available on NCBI Sequence Read Archive SRA under the accession number PRJNA1098702.
