# Supplementary material for: Sediment eDNA metabarcoding reveals the endemism in benthic foraminifera from Arctic methane cold seepages
Source: ISME Commun. 2025 Apr 2;5(1):ycaf058. doi: 10.1093/ismeco/ycaf058 (PMC12700162; doi:10.1093/ismeco/ycaf058)
Supplement: Figure_S1_ycaf058 [file figure_s1_ycaf058.pdf]

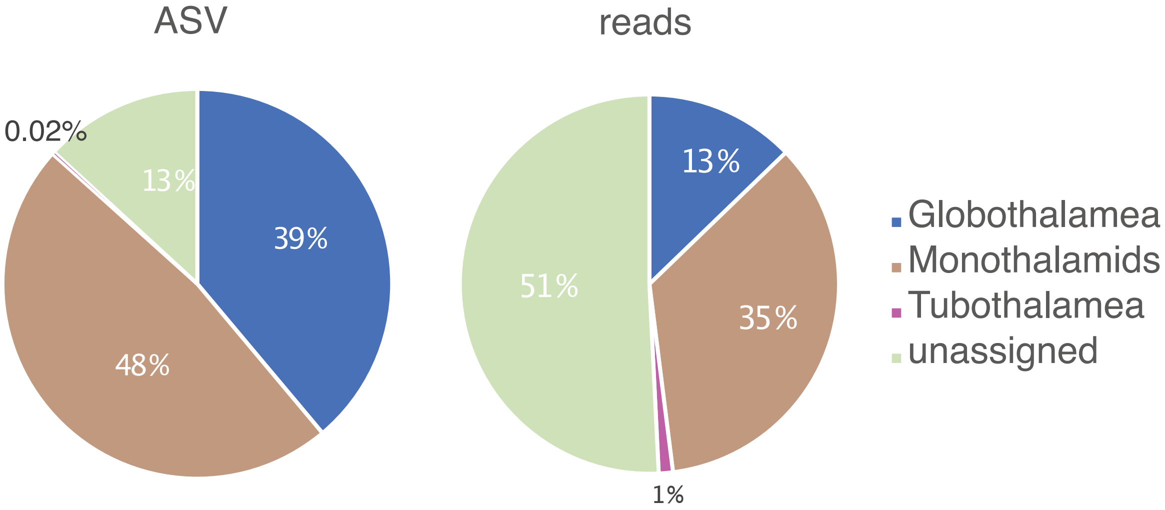


**Figure S1**: General overview of obtained foraminiferal sequences at higher taxonomic rank in percentage of ASV and reads.
