# Supplementary material for: Sediment eDNA metabarcoding reveals the endemism in benthic foraminifera from Arctic methane cold seepages
Source: ISME Commun. 2025 Apr 2;5(1):ycaf058. doi: 10.1093/ismeco/ycaf058 (PMC12700162; doi:10.1093/ismeco/ycaf058)
Supplement: Figure_S2_ycaf058 [file figure_s2_ycaf058.pdf]

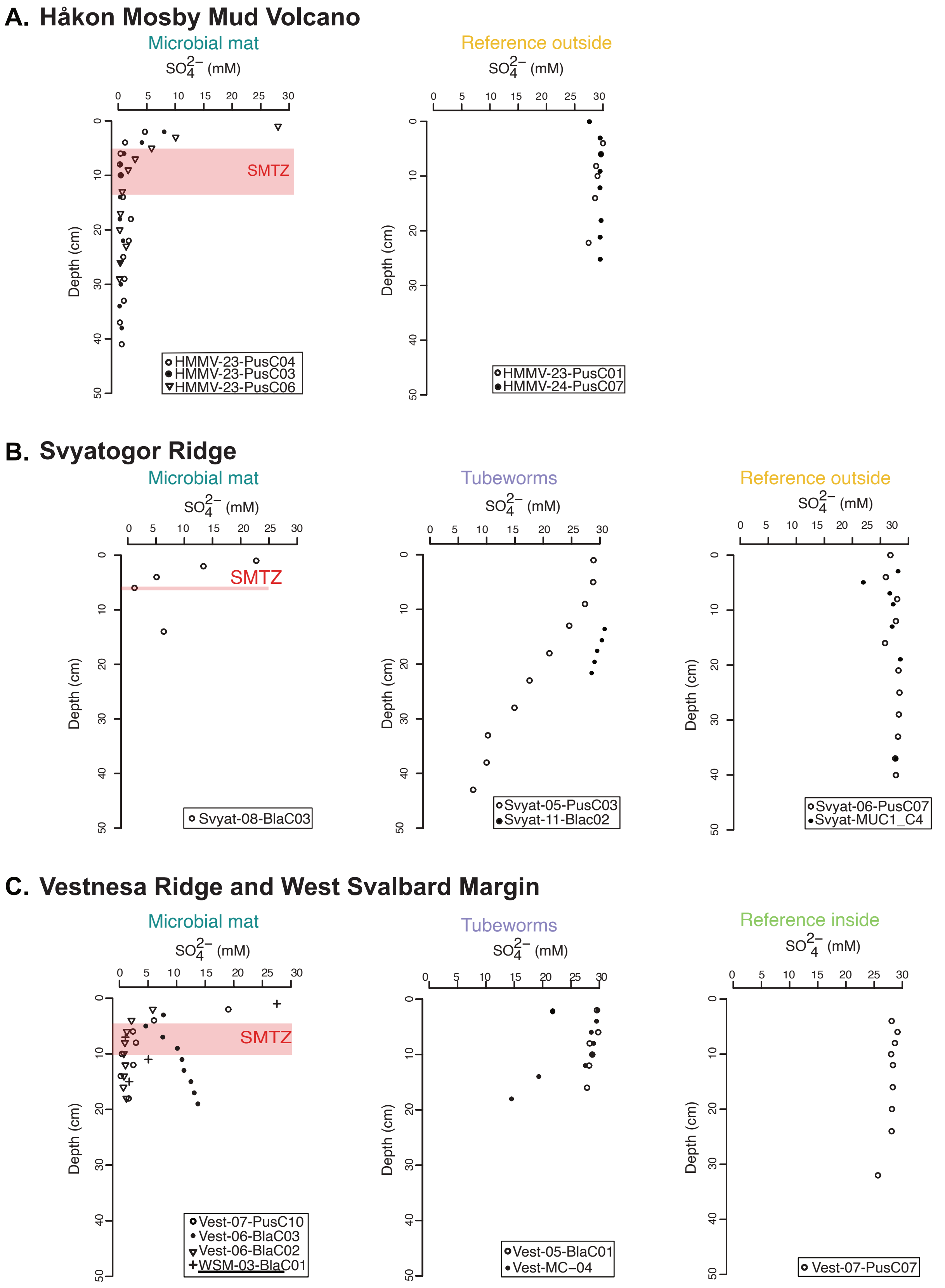

Figure S2: Sulfate profiles: Down-core sulfate concentration profiles across different microhabitats revealed shallow SMTZs in microbial mats centered at around 6 to 12 cm depth. A gentle decrease in sulfate concentration was also observed downwards in the tubeworm microhabitats, while reference sites, both inside and outside pockmarks, showed minimal depth-related changes. **A.** In HMMV (Fig. 2A), microbial mat microhabitats indicated SMTZs at ~6 cm and 10 cm depth. The highest sulfate flux was 260 nmol cm^{-2} d^{-1}, whereas the reference core (HMMV-23-Pusc01) demonstrated a low gradient of 19 nmol cm^{-2} d^{-1}. **B.** At Svyatogor (Fig. 2B), the SMTZ occurred at ~6 cm with a diffusive flux of 290 nmol cm^{-2} d^{-1}. At 14cm, the sulfate increased most likely due to seawater infiltration. Tubeworm microhabitats exhibited gentle sulfate slopes, with Svyat-05-Pusc03 yielding a flux of 88 nmol cm^{-2} d^{-1}. Reference cores displayed flat profiles, indicating negligible sulfate consumption and a low flux of 5 nmol cm^{-2} d^{-1}. **C.** In Vestnesa and WSM (Fig.2C), sulfate was consumed in the top 5–6 cm, marking SMTZs at this depth, with flux of 272 nmol cm^{-2} d^{-1} for core Vest07-PusC10. Tubeworm microhabitats reflected weaker gradients and no clear SMTZ (~20 cm). Vest MC-04 showed an inflection at 12 cm, transitioning to higher gradients and a flux of 161 nmol cm^{-2} d^{-1}. The reference core exhibited a flat gradient, with only the 32 cm depth showing a low flux of 45 nmol cm^{-2} d^{-1}.
