# Supplementary material for: Sediment eDNA metabarcoding reveals the endemism in benthic foraminifera from Arctic methane cold seepages
Source: ISME Commun. 2025 Apr 2;5(1):ycaf058. doi: 10.1093/ismeco/ycaf058 (PMC12700162; doi:10.1093/ismeco/ycaf058)
Supplement: Table_S2_ycaf058 [file table_s2_ycaf058.docx]

**Table S2**. Metabarcoding sequencing data. Reads and ASV counts after LULU curation and by higher taxonomic rank. The final dataset indicates the number of reads and ASV used for further analysis including taxonomic composition, alpha and beta diversities.

|  | **Reads** | **ASV** |
| --- | --- | --- |
| Total after LULU curation | 3,378,032 | 2,054 |
| **Globothalamea** | 1,302,116 | 187 |
| - Planktonic species | 19,212 | 10 |
| **Monothalamids** | 1,605,066 | 578 |
| **Tubothalamea** | 6,145 | 19 |
| **Unassigned** (with Foraminifera genetic pattern) | 464,705 | 1,270 |
| - ASVs with < 100 reads |  | 849 |
| - ASVs with > 100 reads |  | 66 |
| Final dataset for analysis (> 100 reads) | 3,071,522 | 432 |
